# Unveiling the metaphors of (in)formal care: understanding dementia through language

**DOI:** 10.1093/geront/gnaf194

**Published:** 2025-09-11

**Authors:** Greta Rizzi, Anna Messina, Rebecca Amati, Anna Maria Annoni, Emiliano Albanese, Maddalena Fiordelli

**Affiliations:** Institute of Public Health, Faculty of Biomedical Sciences, Università della Svizzera italiana, Lugano, Switzerland; Institute of Public Health, Faculty of Biomedical Sciences, Università della Svizzera italiana, Lugano, Switzerland; Institute of Public Health, Faculty of Biomedical Sciences, Università della Svizzera italiana, Lugano, Switzerland; Institute of Public Health, Faculty of Biomedical Sciences, Università della Svizzera italiana, Lugano, Switzerland; Institute of Public Health, Faculty of Biomedical Sciences, Università della Svizzera italiana, Lugano, Switzerland; Institute of Public Health, Faculty of Biomedical Sciences, Università della Svizzera italiana, Lugano, Switzerland

**Keywords:** Qualitative content analysis, Informal caregivers, Caregiver–person with dementia relationship, Disease experience

## Abstract

**Background and Objectives:**

Metaphorical language is often used to articulate the complex nature of care and can provide valuable insights about it. By analyzing the language of formal and informal caregivers of people with dementia, we aimed to understand their conceptualization of the disease, their role, and their relationship with the patient.

**Research Design and Methods:**

We conducted a study of six focus groups with caregivers of people with dementia (*N* = 6 formal; *N* = 13 informal) as part of the Swiss adaptation of the World Health Organization *iSupport for Dementia* program.

We ran a qualitative content analysis on metaphors caregivers used to talk about dementia, their experience, and their relationship with the person affected. The analysis included metaphors identification, meaning and linguistic complexity analysis, and contextual interpretation.

**Results:**

Caregivers frequently referred to people with dementia as “children” or employed natural elements to depict their burden. Metaphors reflected different aspects of caregiving. Some highlighted an individualistic perspective of maintaining independence while “patching” problems or “drowning” in challenges; others introduced a community-oriented perspective of moral dedication, such as the “mission” metaphor; others focused on the power and dependence dynamics in the person with dementia–caregiver relationship.

**Discussion and Implications:**

While common metaphors in the discourse on dementia were absent, contextual and temporally conditioned metaphors were present. Metaphors collectively provided a multifaced view of caregiving, presenting perspectives of tension between self-care and service to others.

Understanding the plurality of caregivers’ experiences through metaphors can enhance the caregivers–professionals communication, improve care quality, and help address stigma and misconceptions about dementia.

## Background and objectives

### Dementia caregiving

As of 2024, approximately 153,000 individuals in Switzerland are living with dementia, a figure projected to more than double by 2050 (Alzheimer Svizzera, 2024). Most people with dementia live at home, where they are cared for by formal and informal caregivers. The former are usually healthcare professionals or trained personnel who provide paid, structured, and scheduled care as part of their professional duties. Instead, informal caregivers are typically family members or next of kin who provide care without any financial compensation, and often no formal healthcare training or support (Messina et al., 2022). The burden of caregiving for people with dementia falls predominantly on informal caregivers rather than formal caregivers and support systems. In Switzerland, for each person living with dementia, there are on average three informal caregivers, who bear nearly 50% of the total costs of dementia care (Alzheimer Svizzera, 2024).

Regardless of the type of care, caring for people with dementia is challenging, physically and emotionally strenuous, and is associated with anxiety, depressive symptoms, and poor quality of life (Chiao et al., 2015). Dementia is an incurable neurodegenerative disease, associated with increasingly remarkable care needs (Chiao et al., 2015). Caregivers are compelled to provide increasingly extensive support, from basic activities of daily living (ADLs) such as personal care (e.g., bathing, dressing, etc.) to instrumental activities such as managing financing and medication and constant supervision. Integrating care between formal and informal caregivers is essential for delivering holistic, continuous, and comprehensive support, which is vital given the complex needs of people with dementia. Effective integration depends on clear communication, yet this is often hindered by differing role approaches and low recognition of informal caregivers as equal partners in care (Hengelaar et al., 2018).

In Switzerland, a national policy and plan were implemented to respond to the complex individual and societal challenges posed by dementia, with the aim to reduce its burden while ensuring high-quality care for people with dementia and those who care for them (Federal Office of Public Health [FOPH], 2021). Key objectives of this strategy include promoting home-based care and enhancing support to and coordination among the various figures involved in home care (Federal Office of Public Health [FOPH], 2021). However, despite abundant research on caregiving burdens (Van Den Kieboom et al., 2020), we still have limited insight into the nuanced, unique experiences of caregivers throughout the care process. Little attention has been given to exploring the valuable and revealing perspectives they offer (Messina et al., 2022).

### Metaphors in dementia

Non-literal/figurative language is often used in the illness discourse to articulate the complex nature of care (Bleakley, 2017; Sontag, 1990; Wohlmann, 2022). Metaphors are non-literal or figurative language commonly used in dialogical contexts, that intentionally require further elaboration compared to literal meaning. According to the Conceptual Metaphor Theory (Lakoff & Johnson, 1980), metaphors are more than descriptive linguistic expressions; they reflect and shape how individuals map, understand, conceptualize, interpret, and communicate about reality—including illness.

In the context of dementia, caregivers may employ voluntary and involuntary non-literal expressions to articulate their emotional and practical struggles. Through metaphors caregivers can communicate complex and abstract experiences by using simple, concrete, and more relatable concepts (Golden et al., 2012), creating a sense of community and giving meaning to the disease. Nevertheless, while enhancing accessibility, metaphors can also distort or oversimplify intricate concepts and phenomena, potentially leading to misunderstanding and stigma (Lakoff & Johnson, 1980; Sontag, 1990). In fact, previous research has shown that metaphors influence how carers approach and treat people with dementia. Some metaphors, such as the warfare metaphor, can be stigmatizing, contributing to dehumanize those affected (George et al., 2016; Sontag, 1990); other metaphors, such as the companion one (Brookes, 2023), can foster a more compassionate perspective, emphasizing the value of moments of lucidity and connection (­Swinnen & Schweda, 2015). Therefore, the analysis of metaphorical language can reveal underlying beliefs about the nature of dementia, its causes, and impact on caregivers’ roles (Golden et al., 2012; Wiersma et al., 2025) and may contribute to inform tailored support strategies as well as to address stigma.

However, despite the growing body of literature on non-literal language in dementia, which includes people with dementia’s narratives (Castaño, 2020; Miyamasu, 2024) and disease representations analysis (Brookes, 2023; Zeilig, 2014), only few studies have analyzed metaphors as a lens in the caregivers’ perspective (Oerlemans et al., 2023; Zimmermann, 2017). These studies suggest that metaphors and their interpretation vary across cultural contexts (Oerlemans et al., 2023), as cultural norms profoundly shape metaphorical thinking and expressions (Kövecses, 2015) and can influence the individual and collective perception of both dementia and caregiving. We conducted this study to expand and advance knowledge on metaphors and dementia. Given the cultural specificity of metaphors, we concentrated our investigation in the Italian-speaking southern region of Switzerland. By analyzing non-literal language in the narratives of formal and informal caregivers of people with dementia in this region, we sought to understand the cognitive and emotional frameworks that shape their conceptualization of the disease, their role, and their relationship with the person affected.

## Research design and methods

The *Materials and Methods* section of this study is presented based on the Consolidated Criteria for Reporting Qualitative Research (COREQ) standard and recommendations for interviews and focus groups (Tong et al., 2007).

### Study design

This is a qualitative descriptive study conducted as part of the Swiss adaptation of the World Health Organization (WHO) *iSupport for Dementia* program (Messina et al., 2022; World Health Organization, 2019).

### Study participants and recruitment

The target population of the study were formal and informal caregivers of people with dementia. Eligible subjects were individuals who (1) were currently or have been in the past caregivers of a person with dementia; (2) fluently spoke Italian; and (3) lived in the canton of Ticino, Southern Switzerland.

The recruitment phase and study’s procedures are described elsewhere (Messina et al., 2022). Briefly, a snowballing technique was used to recruit a convenience sample of caregivers of people with dementia between May and June 2021. Next, we provided to interested caregivers the informed consent document 2 weeks before data collection with information on the study’s nature and scope, focus groups’ audio-recording, and the possibility to withdraw from the study at any moment. Upon receipt of the signed consent form, we used the REDCap (Research Electronic Data Capture) (Harris et al., 2009, 2019) platform to gather participants’ sociodemographic data, information about the relationship of the caregiver and the person with dementia, the status of the person with dementia, and the duration of the caregiving experience.

### Data collection

We conducted focus groups with formal and informal carers of people with dementia to collect data. Widely used in participatory health research (Duea et al., 2022), focus groups leverage social interactions to explore collective perspectives, attitudes, and beliefs, fostering the acquisition of rich and meaningful data. Focus groups were all moderated by a doctoral student and psychologist (A.M.), supervised by team members with experience in qualitative methods (M.F., R.A.).

The sessions had a structured format: first, caregivers introduced themselves and shared their personal experiences. Then, a comprehensive discussion on the *iSupport for Dementia* ­program for caregivers (World Health Organization, 2019) ensued. A specific module of the program (World Health Organization, 2019) and related scenarios were addressed in each focus group. Specifically, we discussed about communication with people with dementia (module 2 of the program), caregivers’ well-being (module 3), provision of daily care, including hygiene and nutrition (module 4), and the management of behavioral and mood changes (module 5). Participants were invited to reflect on the relevance and familiarity of scenarios representing everyday examples of caregiving. Throughout the discussions, we observed that they frequently employed metaphorical language to articulate and share their personal experiences. This observation led us to conduct a more in-depth analysis of the metaphors used.

### Data (transcription, translation, and) analysis

Focus groups were audio-recorded and transcribed verbatim by an independent research assistant. During transcription, a quality control check of the transcripts was performed, and sensitive personal information was pseudonymized to ensure privacy.

We performed an inductive content analysis (Steger, 2007) focused on the non-literal/figurative expressions that caregivers used to talk about dementia and their experience. By non-literal/figurative expressions we mean here only metaphor and similarities, which use comparison between two terms to convey additional information at the literally expressed concept.

The content analysis was structured in three steps, integrating methods from the Grounded Theory for qualitative analysis (Glaser & Strauss, 2017) and Steger’s approach (Steger, 2007) for metaphors in narratives’ analysis and interpretation:

1. We read focus groups’ transcripts carefully to familiarize ourselves with data and get an overview of the metaphors’ use and their distribution in the text (Steger, 2007). We identified metaphors in the text (Steger, 2007) and developed an initial open coding taxonomy of concepts (Glaser & Strauss, 2017). Two members of the research team (M.F., G.R.) compared and agreed on the metaphors used to depict the caregivers’ personal experience and dementia, excluding those expressions pertaining to speech orientation that were not relevant to the purpose of this study.
2. We divided metaphors into interpretative semantic categories based on the relationship between concepts in the open coding taxonomy and debated the meaning attributed to the metaphor in common sense and in the literature, as well as by the cultural context (Steger, 2007).
3. Finally, we interpreted the meaning (i.e., metaphors tell us about caregivers’ experiences and emotional states) and implications of the metaphors (i.e., how these insights could inform support strategies or policy-making) in the context of caring for people with dementia (Steger, 2007). The research team members reached consensus about the expressions’ interpretation.

### Artificial intelligence–assisted technology

During the preparation of this work, the author(s) used ChatGPT/OpenAI in order to improve readability. The author(s) reviewed and edited the content as needed and take(s) full responsibility for the content of the publication.

## Results

Between May and August 2021, we conducted a total of six focus groups with 19 carers of people with dementia. We held five in-person focus groups with informal caregivers, while one remote focus group with formal carers to allow them to participate according to their needs and anti-COVID-19 measures. Focus groups lasted 2 hr on average, ranging between 110 and 130 min.

### General characteristics of participants

A total of 19 caregivers participated in the focus groups; 13 (68.42%) were informal caregivers only, 3 (15.79%) were formal caregivers with professional experience in dementia caring, and 3 (15.79%) were formal carers who reported being or having been in the past informal caregivers. Most caregivers were women (*N* = 16, 84.21%). Group sizes varied between 2 and 8 participants per session, with most individuals attending multiple focus groups, resulting in diverse group compositions across sessions. Sociodemographic characteristics of study participants are presented in Table 1.

**Table 1.** Sociodemographic characteristics of caregivers

Participant ID	Formal vs informal caregiver	Sex	Age (years)	Employment status	Years of personal caring experience	Years of professional caring experience	Relationship with the patient with dementia
**1**	Formal + informal	Female	52	Housewife/retired	>10	>10	N.A.
**2**	Formal + informal	Female	54	Housewife/retired	>10	>10	N.A.
**3**	Formal	Female	28	Housewife/retired	0	6–10	N.A.
**4**	Formal + informal	Female	45	Employed	<1	<1	N.A.
**5**	Formal	Female	29	Housewife/retired	0	1–2	N.A.
**6**	Formal	Female	59	Employed	0	>10	N.A.
**7**	Informal	Female	58	Housewife/retired	3–5	N.A.	Spouse
**8**	Informal	Female	55	Housewife/retired	3–5	N.A.	Son/daughter
**9**	Informal	Female	59	Housewife/retired	3–5	N.A.	Spouse
**10**	Informal	Male	67	Employed	3–5	N.A.	Son/daughter
**11**	Informal	Female	58	Housewife/retired	1–2	N.A.	Spouse
**12**	Informal	Male	57	Employed	3–5	N.A.	Son/daughter
**13**	Informal	Male	74	Employed	6 to10	N.A.	Spouse
**14**	Informal	Female	55	Employed	3–5	N.A.	Son/daughter
**15**	Informal	Female	75	Housewife/retired	>10	N.A.	Son/daughter
**16**	Informal	Female	76	Housewife/retired	3–5	N.A.	Spouse
**17**	Informal	Female	82	Housewife/retired	1–2	N.A.	Spouse
**18**	Informal	Female	55	Employed	1–2	N.A.	Son/daughter
**19**	Informal	Female	81	Housewife/retired	3–5	N.A.	Spouse

*Note.* N.A. = not applicable.

### Main results

In the narratives of formal and informal caregivers of people with dementia, we identified and organized metaphors into three major categories presented in the following paragraphs:

- metaphors referring to the person with dementia or to the disease.
- metaphors referring to being a caregiver of a person with dementia.
- metaphors referring to the relationship between the person with dementia and the caregiver.

**Figure 1.**
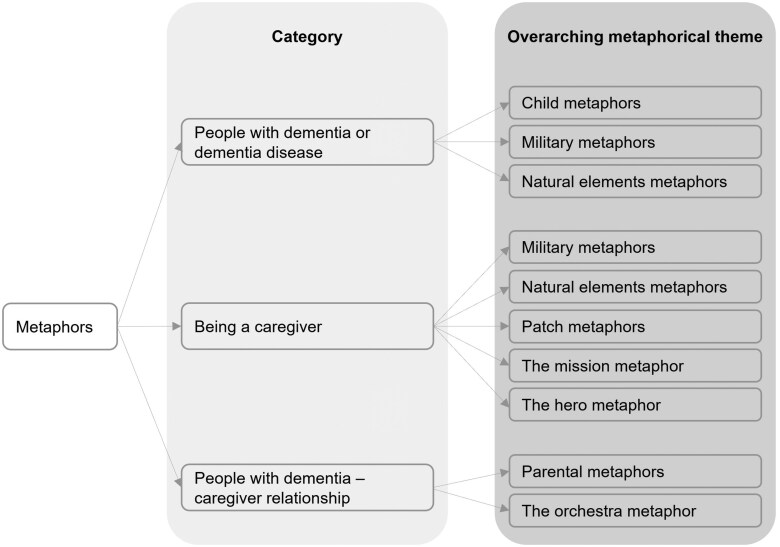
Overview of the metaphorical overarching themes by category.

In each category, we grouped results in semantically defined themes, i.e., terms such as “battle” or “defeat” semantically refer to the concept of war and fall under the military theme. A comprehensive overview of the overarching themes by category emerged in our focus groups and is presented in Figure 1.

#### Metaphors referring to people with dementia or dementia disease

##### Child metaphor

Formal and informal caregivers commonly likened people with dementia to children, a phenomenon known as “infantilization” in psychology (Marson & Powell, 2014). The metaphor highlighted decline in cognitive and functional abilities of people with dementia, and behaviors reminiscent of childhood, to which a regressive mechanism may be attributed.

I take care of a seventy-year-old lady who is actually like a seven-year-old; she is truly a child; we must accompany her even in the daily actions: eating, getting up, dressing, reminding her to brush her teeth… just the basic things you teach children. (P.3, female, formal carer)

The child metaphor portrayed individuals with dementia as overly dependent on others, neglecting their ability to participate in daily activities, their past experiences, and identity.

However, despite the prevalent use of infantilization, some caregivers acknowledged the persistence of long-established personality traits in people with dementia, suggesting that their identity is not entirely erased and that “we shouldn’t forget, there is some resentment of the past, lived life” (P.9, female, informal caregiver). In this regard, an informal caregiver noted differences in how her parents react to their condition and care: she shared the observation of her father’s joyful, childlike wonder, alongside his obedience, in contrast to the non-cooperative, resistant personality of her mother, which made caregiving more challenging.

In some ways, my father has become like a child again, as he is joyful and cheerful, genuinely amazed by things. […] Then, my father is an obedient child, let’s say, while my mother is not. (P.18, female, informal carer)

##### Military metaphors

Most informal caregivers used terms of military derivation such as “battle,” “defeat,” or “fronts,” to talk about the disease. Dementia has been described as a “battle” with a definite outcome: there is no chance of victory, as suggested by an informal carer:

It was an escalation of the disease’s problems, and you can’t do anything; for me, this is truly the worst thing. There is nothing to be done, it’s a lost battle from the start. And I experienced it in all its phases until […] I had to take her to a special home for (people with) Alzheimer’s disease. (P.13, male, informal carer)

##### Natural elements metaphors

In this category, we grouped metaphors that draw parallels between dementia and different natural elements, which evoked different characteristics and manifestations of the disease.

Dementia has been compared with a “wave” to emphasize the fluctuating nature of the condition, mirroring daily variations in cognitive functioning, ranging from clarity to disorientation, and agitation interspersed with calm.

In my opinion, it is really the disease that is different every day. […] And so it is really a wave. (P.8, female, informal carer)

Additionally, an informal carer occasionally used the metaphor of “living in a bubble” to highlight a detachment from a shared reality and the dwindling ability to significantly interact with others. The bubble, while isolating from external connections, is something carers strive to maintain to shield people with dementia from the distress of recognizing their limitation, preserving their dignity and inner peace.

I phone her every evening, and I visit her every week, but she lives in her bubble. It is true that she is 96 years old, and she lived her life, but I wait only for the moment (she dies.). (P.10, male, informal carer)

#### Metaphors referring to being a caregiver of a person with dementia

##### Military metaphors

In addition to the nature of the disease, the metaphor of the “lost battle” was also commonly used by informal carers to express the helplessness and frustration experienced when taking care of a loved one with dementia, but also a desire to not surrender.

I will volunteer in Alzheimer’s centers because I feel compelled to also give others what I gave to my wife, with the sadness that it is a lost battle. (P.13, male, informal carer)

In this perspective, caregiving has been represented as an emotional battle as the carer suffers from seeing the person affected gradually and inexorably lose cognitive abilities and autonomy, but also an implicit allusion that at the moment there is no cure.

##### Natural elements metaphors

Caregivers have most frequently referred to the sea, which resonated in speeches with terms such as “waves” or “drowning,” to describe their experience; though, they used it with different meanings. On the one hand, the “wave” has been described by a caregiver with a more positive connotation when referring to a surface with two sides that reflect two different approaches to the role of caregiver.

It is really a wave…a sea wave that perhaps would be good to look at on the other side. (P.8, female, informal carer)

On the other hand, the metaphor of “drowning” expressed the perception of being overwhelmed by the emotional, physical, and mental challenges of caring for a person with dementia, conveying feelings of burden, stress, and frustration. Some also likened this feeling to “being in a vortex.”

Deeper information on the causes of this burden came from the metaphor of “an unfathomable magma.” Magma usually refers to something boiling, ready to explode and potentially destructive; in the dementia context, it may parallel the degenerative and unpredictable changes in the person affected. Describing it as “unfathomable” sheds light on the caregivers’ difficulty in understanding what the person with dementia feels and perceives about of his/her own condition, including the ability to perform activities autonomously.

When the desire to have control over small aspects of their own lives remains, they are very much aware of it… This has become an unfathomable magma. (P.15, female, informal carer)

In addition, the burden can lead to the desire for a “desert island,” that symbolizes a longing for respite and escape from the constant demands of caregiving and a quest for relief from stress and responsibility.

You get out of a situation you can’t take anymore, meaning the only thing you want is the desert island with no one, not even a cell phone, and you are unreachable. (P.14, female, informal carer)

##### Patch metaphor

The “patching” metaphor evokes something damaged that has been repaired with temporary and inadequate solutions. Carers used it to describe the constant attempt to balance caring responsibilities with personal resources and limited time.

The metaphor revealed a caregiver’s sense of guilt about leaving her mother alone during the day, which led her to compensate by dedicating all her free time to the mother’s care, at the expense of personal interests and relationships.

I felt a bit guilty about leaving in the morning to go to work and coming back in the evening, so I tried to patch things up as best as I could. (P.15, female, informal carer)

##### Mission metaphor

Caregiving was also experienced as a “mission.” In this perspective, caring for a family member with dementia is perceived as a task with a moral value, to be performed with sacrifice and unwavering dedication. Caregiving becomes the priority in the life of the carer, an all-encompassing experience at the expense of personal needs and well-being.

It is a life choice because many times one can also say “I cannot do this” or “I don’t feel close enough to this person to ruin my life”; because you ruin your life… that is, you dedicate your life to this mission. (P.13, male, informal carer)

##### Hero metaphor

In the common sense, a hero is a person endowed with extraordinary virtues of courage, strength, and sacrifice. In our focus groups, an informal carer expressed a reluctance to adopt this role, acknowledging personal needs and limitations in providing care for a spouse with dementia. It was noted that the expectation to act heroically can be detrimental to personal wellbeing, particularly as aging leads to a gradual decline in energy.

I think with my mindset, I’ll know when it’s time to make a decision, not to be the hero at all costs. (P.9, female, informal carer)

#### Metaphors referring to the relationship between the person with dementia and the caregiver

##### Parental metaphor

The parental metaphor is closely related to infantilization of people with dementia, but it highlights a change in the family dynamics. In particular, caregivers described a deep role reversal that occurs in the person with dementia–caregiver relationship. Adult children or spouses become actively responsible for the care of their loved ones, providing them with assistance and supervision, even in the simplest daily tasks.

It was difficult for me to accept first of all to be a mother to my mother […]. Now we have been to the sea together and right there (I had to be a mother) 100% because even to go to the bathroom, she always needed help, support, to check that she cleaned. (P.14, female, informal carer)

Change in family dynamics can be emotionally difficult to accept, as the caregiver should adapt to a new role of both authority (“Sometimes, the person with dementia as a child has to be a little contained” P.1, female, formal and informal carer) and comprehension (“It’s like with children: you try to say it in the good manner” P.18, female, informal carer) toward a person who once cared for them. According to a caregiver, such parenting responsibility often falls more heavily on female family members, who face limitations similar to those experienced when caring for kids, such as restrictions on daily activities, and the feeling of being confined to the needs of care.

As I said, you pass from the role of children to that of mother […]. Especially, the woman finds herself with all the limitations of when she raised kids: you cannot do this, you cannot do that …. (P.14, female, informal carer)

##### Orchestra metaphor

The orchestra metaphor depicted the caregiver–person with dementia relationship as a complex power and dependence dynamic. Though mentioned only once, it effectively captured the caregivers strive in balancing care for the patient with their independence. In the parallelism, the person with dementia is likened to an orchestra maestro, assuming a dominant role in the life of the caregiver, whose daily activities are conditioned and subordinated to the needs of the cared one. The subordinate role may lead to an inner control–autonomy conflict for the caregiver.

If you don’t seek help, in a short time you can’t do it anymore […] There is the patient who directs the orchestra, and I am not willing to let him direct my life, but sure I am conditioned…. (P.9, female, informal carer)

**Figure 2.**
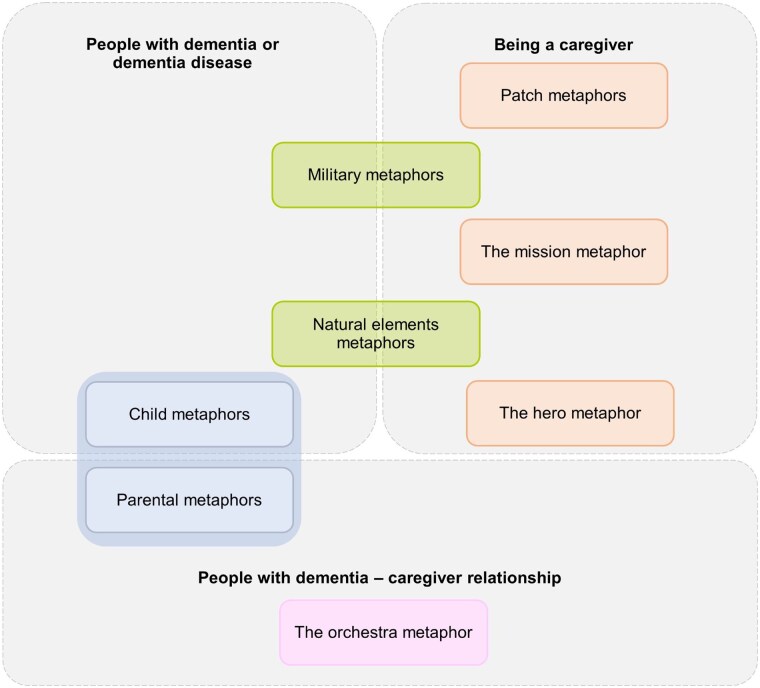
Shared metaphorical semantic themes across categories.

The tripartite categorical organization allowed us to foreground the different dimensions of the caregiving experience, as expressed through metaphors. At the same time, it enabled us to trace recurring semantic patterns that cut across categories, offering additional insight into how caregivers draw on shared metaphorical domains to articulate distinct yet interconnected aspects of dementia and caregiving. Shared thematic patterns across categories are visually summarized in Figure 2.

## Discussion and implications

We explored metaphors used by formal and informal caregivers of people with dementia in the Italian-speaking region of Switzerland. In our focus groups, caregivers often employed metaphors to verbalize complex emotional and experiential realities and to convey vivid descriptions of their feelings. Figurative language helped carers to communicate their perceptions about the disease and those affected (child metaphor, military metaphors, and natural elements), their role (military metaphors, natural elements, patch, mission, and hero), and their relationship with people with dementia (parental and orchestra metaphors).

Overall, our findings are only partially aligned with the existing literature on the metaphors used in dementia discourse.

First, one of the most notable aspects of our study was the consistent use of the child metaphor to characterize people with dementia. Infantilization traditionally reflects cognitive and behavioral decline with resemblance to a childlike state, where guidance and supervision similar to parental care are required (Berdes & Eckert, 2007; Lövenmark, 2020; Thorsen & Johannessen, 2021). As already debated in the literature, this framing that tends to highlight the contrast between the individual’s functional past and current state (Jongsma & Schweda, 2018; Wanka et al., 2024), may be limiting and reinforce misconceptions about dementia (Zimmermann, 2017). Viewing people with dementia through the lens of infantilization neglects the personal identity they developed and expressed over the life-course and oversimplifies dementia to a mere regression state, rather than recognizing it as a complex condition that continues to involve the individual’s history and personality (Wanka et al., 2024). Also, carers may unconsciously adopt a parent-like role to fulfill the attachment needs of the person with dementia, such as dependence, emotional vulnerability, and reduced self-regulation (Miesen, 2014). But, assuming a paternalistic role, caregivers may strip people with dementia of their autonomy, leading to a diminished perception of their agency. This reinforces social stigma that associate dementia with incompetence and helplessness (Wanka et al., 2024).

Despite infantilization was still prevalent in our narratives, some informal caregivers used the “child” metaphor with a nuanced dimension. Rather than simply equating individuals with dementia to children expected to become autonomous and develop their own identity in the future, informal caregivers acknowledged the uniqueness of this phase, suggesting that individuals with dementia maintain distinct personal traits and modes of expression. This perspective reflects a deeper recognition of their individuality and foster both a more empathetic and respectful view of affected people as well as positively influence caregiving strategies, simplify interactions, or comfort the individual (Berdes & Eckert, 2007; Chung et al., 2017).

Second, as common in the illness discourse (Parsi, 2016; Wohlmann, 2022; Zimmermann, 2017), metaphors related to military contexts were prevalent throughout the narrative corpus. Such metaphors were mainly used to describe the neurodegenerative nature of the disease or the daily challenges of providing care for people with dementia. Unlike previous studies on both people with dementia and caregivers, we did not find a personification of the disease as a cruel actor, an enemy, or an invader stealing individuals’ functionality (Brookes, 2023; Marhánková & Honelová, 2024; Miyamasu, 2024). Our caregivers did not attribute a malevolent intent to the disease; war-related metaphors described personal struggles or challenges to cope with (Wohlmann, 2022), rather than being directed toward an external aggressor. This could be attributed to the Swiss historical neutrality policy. Indeed, despite military service is part of the national fabric, making military language accessible and understandable in the common consciousness, the Swiss army serves as self-empowerment instrument rather than an instrument of aggression toward a defined enemy.

Third, in our focus groups, metaphors related to natural elements were frequently evoked, mainly reflecting feelings of vulnerability and uncertainty associated with managing people with dementia in the face of the disease’s progression. To the best of our knowledge, these metaphors have only been little represented in studies so far (Castaño, 2020). The prominence of environmental metaphors in our study may be ascribed to the so-called availability heuristic, according to which people tend to draw on readily accessible knowledge when constructing their understanding of a situation. In this context, nature-related concepts may be particularly salient due to their increased presence in contemporary discourse. In recent years, the increased global focus on environmental issues, such as climate change and sustainability, may have made nature-related concepts more pervasive in everyday thought and language. Furthermore, as metaphors are strongly influenced by the context (Kövecses, 2015; Lakoff & Johnson, 1980 ), geographical factors may have also played a role. In southern Switzerland, where individuals are regularly exposed to expansive green and blue spaces (Chênes et al., 2021), natural elements are often familiar, commonplace, and deeply integrated into daily life.

Moreover, compared to previous studies that typically presented the caregiver role as a source of burden (Cabote et al., 2015; Chiao et al., 2015; Lövenmark, 2020), our research emphasizes the coexistence of multiple perspectives offering a complex representation of caregiving. On the one hand, a more individualistic and self-centered view of caregiving emerges from metaphors such as the “orchestra” and the “hero,” which point up to the importance of providing care commensurate with one’s abilities and preserving self-determination, without being subordinated to the needs of people with dementia. On the other hand, beyond the recognized focus on individualistic behaviors (Brookes, 2023; Peel, 2014), our study also represents a community-oriented and altruistic perspective on caregiving (Pyke & Bengtson, 1996). This perspective is reflected in the desire to support other carers, while acknowledging the inherent challenges of the role, as captured by the metaphor of the “lost battle.” The battle metaphor extends to the reluctance to surrender in spite of defeat, and by the empathetic and altruistic attitude toward other, non-relative, people with dementia after the personal battle is over. Likewise, this attitude is conveyed through metaphors that evoke a sense of purpose and dedication, such as caregiving as a “mission.” Though mentioned by only one carer, this unique conceptualization frames caregiving not merely as a set of tasks, but as a profound commitment that transcend daily assistance and permeates the carers’ life, reflecting not just personal obligation but a wider, altruistic vision. This may echo societal influences, such as the strong religious tradition in the region, as well as legal frameworks in Switzerland that promotes home care, emphasizing the duty and importance of caregivers within the family unit and the community. However, a home care-oriented society may contribute to generate the expectation of altruistic behaviors, which can in turn have negative implications. Indeed, caregivers’ frustration, that is widely regarded as the most distressing aspect of care, tends to escalate as the disease advances and reaches its peak when carers recognize they can no longer manage the demands of home care, as if they had failed to accomplish their “mission.”

### Limitations

Some limitations of this study are worth noting. First, our study was part of a larger research endeavor which provided the opportunity to analyze metaphors used by caregivers of people with dementia (Messina et al., 2022, 2024). By design, the study was not originally intended to elicit in-depth caregivers’ narratives, but the conversational context encouraged participants to share their experiences. We did not prompt neither did we explicitly ask caregivers to describe their experiences with non-literal expressions; however, metaphors were commonly and spontaneously used. The natural use of metaphors likely enhanced the authenticity of the data, aligned with best practices in participant-centered qualitative methodology (Vaughn & Jacquez, 2020), and minimized researcher-induced bias.

Second, our sample consisted mainly of female informal carers. While the sex distribution of the sample reflects statistical data showing that females are more likely to assume caregiving roles (Whitten, 2022), it would be interesting to investigate whether and to what extent metaphors differ according to gender.

Similarly, formal caregivers were underrepresented in our study sample. Notably, their narratives exclusively relied on infantilization to describe people with dementia. Resilience on a single metaphor may reflect two aspects: first, an emotional involvement that differs from that of informal carers, as it is not shaped by familial or friendship dynamics; second, a homogeneous approach to care (Dooley et al., 2015) rooted in professional training. In this case, it is worth noting that if formal caregivers are trained to approach people with dementia as children, this challenges the principles of person-centered care (Wanka et al., 2024). Such an approach risks depriving individuals of their autonomy, reducing their active participation in care, and increasing the likelihood of resistance to it (­Jongsma & Schweda, 2018). However, given the limited number of formal carers in our sample, observations made about infantilization in this subgroup of participants should be considered as inferences from data and cannot be generalized. Further exploration of the metaphors used by formal caregivers could offer deeper insights into how professionals and informal carers conceptualize their roles differently, thereby facilitating dialogue and understanding between these groups.

### Implications

Metaphors are widely used in medical discourse by physicians, caregivers, and patients (Sontag, 1990), particularly in the context of life-altering conditions such as cancer, cardiovascular diseases, and chronic, noncurable diseases (Bleakley, 2017), such as Alzheimer’s (Golden et al., 2012). In dementia discourse, metaphors hold critical implications for both clinical practice and public understanding, serving as a communicative bridge between informal carers and professionals while shaping broader societal narratives about the disease.

In healthcare settings, professionals use metaphors to simplify medical concepts and build trust, often with a more neutral and goal-oriented tone (Bleakley, 2017). For caregivers and patients, metaphors may provide a means to describe their experiences and express emotions in a more relatable and accessible way (Bleakley, 2017; Zimmermann, 2017), offering a creative outlet for feelings that might be difficult to articulate with direct language. In the context of caregiving, the use of metaphors is peculiar because of the dual nature of the role: caregivers are both daily observers of the disease and active participants in care. Our study confirms that the use of metaphors by caregivers of people with dementia is deeply personal and emotionally charged. The dual nature of the role is embedded in the metaphors caregivers choose, which encapsulate their conceptualization of the disease, coping mechanisms, contextual influences, and the interplay between personal resilience and shared suffering and empathy with the person with dementia. This plurality of perspectives—ranging between self-care and service to others—though different, co-exists and contributes to delineate an articulated understanding of the caregiving experience. Therefore, appraising metaphors’ communicative power can significantly improve communication between caregivers and professionals and inform programs that resonate more closely with caregivers’ lived experiences (Wiersma et al., 2025).

While metaphors can simplify technical jargon and enhance communication in healthcare, their interpretative flexibility requires caution as the widespread use of certain metaphors—such as war-related and infantilizing metaphors—may have unintended consequences on care delivery (George et al., 2016; Sontag, 1990; Wanka et al., 2024). The same metaphor may hold different meanings depending on the context: for some, war metaphors may signify resilience and determination, while for others, they may evoke stress and exhaustion (Sontag, 1990; Wohlmann, 2022). Similarly, an altruistic metaphor like caregiving as a “mission” can either highlight dedication and inform the implementation of programs emphasizing companionship or reinforce unrealistic expectations of self-sacrifice, leading to increased burnout. The risk lies in the uncritical acceptance of metaphors as neutral descriptors rather than as subjective, culturally influenced constructs that reflect deeper cognitive structure (Lakoff & Johnson, 1980). To address this issue, caregivers should be informed on the importance of communicating their needs consciously and professionals encouraged to ask structured questions that can help disambiguate the meaning behind non-literal expressions used by caregivers. Understanding caregivers’ perspective and needs is the starting point to draw targeted approach and shape support systems that are not only relevant but also effective in addressing the challenges of caregivers.

There are potential implications also for formal healthcare education, as metaphors also shape care environments. The frequent infantilization of people with dementia challenges the principles of person-cantered care and may lead to overly paternalistic caregiving (Jongsma & Schweda, 2018). Formal caregivers, trained within a system that implicitly reinforces such metaphors, may unconsciously adopt care practices that prioritize control over autonomy. Therefore, professional training programs should equip formal carers with the communication skills necessary to use language thoughtfully, promoting alternative frameworks that uphold the dignity, individuality, and lived experiences of people they care for (Chung et al., 2017).

Finally, beyond clinical settings, metaphors used in public discourse play a pivotal role in shaping societal attitudes toward dementia (Zeilig, 2014) and can have implications for public awareness and stigma. While metaphors can demystify dementia and foster empathy in the broader public (Wohlmann, 2022), they can also reveal a reduced public understanding and reinforce misconceptions (Zimmermann, 2017) that undermine efforts to improve care quality. Public health campaigns and media representations should judiciously assess the metaphors they employ to promote a respectful understanding view of dementia.

## Conclusions

Our study on the metaphors used by caregivers of people with dementia to describe their experiences provides a comprehensive view of caregiving. It acknowledges not only the difficulties and stress related to caregiving but also the altruistic perspective of providing care. Some common metaphors in the discourse on dementia did not emerge, other metaphors, like the warfare and the metaphor of the child, were frequently used by our participants, sometimes with nuanced interpretations compared to existing literature. The metaphors spontaneously invoked by caregivers in their narratives were shaped by the societal context and influenced by time; as for the case of framing caregiving as a mission or comparing it to natural phenomena. Our study is a bold demonstration of the fact that metaphors are not only a linguistic tool but also a psychological and social resource that enriches how caregivers express their experiences and connect with others. Given this, it is important to recognize the dual scope of metaphors—their ability to both illuminate and distort meanings—and carefully analyze their use in discussions about dementia. By fostering awareness of metaphor use, we can encourage a discourse in which healthcare professionals acknowledge and value caregivers’ experiences as well as ensure that people with dementia are seen as individuals with agency and a continued sense of self, ultimately improving quality of care, and support systems for caregivers.

## Data Availability

Anonymized data used to conduct the present study will be made available upon reasonable request from the corresponding author. Data were not pre-registered.
